# Prenylated Isoflavonoids-Rich Extract of Erythrinae Cortex Exerted Bone Protective Effects by Modulating Gut Microbial Compositions and Metabolites in Ovariectomized Rats

**DOI:** 10.3390/nu13092943

**Published:** 2021-08-25

**Authors:** Hui-Hui Xiao, Xueli Yu, Chen Yang, Chi-On Chan, Lu Lu, Sisi Cao, Siu-Wai Wan, Ze-Jun Lan, Daniel Kam-Wah Mok, Sheng Chen, Mansau Wong

**Affiliations:** 1 State Key Laboratory of Chinese Medicine and Molecular Pharmacology (Incubation), Shenzhen Research Institute, The Hong Kong Polytechnic University, Shenzhen 518057, China; huihui.xiao@polyu.edu.hk (H.-H.X.); yuxueli_111@163.com (X.Y.); on.chan@polyu.edu.hk (C.-O.C.); llluuuiii@126.com (L.L.); daniel.mok@polyu.edu.hk (D.K.-W.M.); 2 Department of Applied Biology and Chemical Technology, The Hong Kong Polytechnic University, Hong Kong 999077, China; caosisi0703@gmail.com (S.C.); siuwai.wan@polyu.edu.hk (S.-W.W.); shechen@cityu.edu.hk (S.C.); 3 College of Animal Science and Technology, Nanjing Agricultural University, Nanjing 210095, China; cyang69-c@my.cityu.edu.hk; 4 Medical School, Shenzhen University, Shenzhen 518067, China; hardboy265@163.com

**Keywords:** Erythrinae cortex, gut microbiota, short chain fatty acids, prenylated flavonoids, ovariectomy rats

## Abstract

Flavonoids, found in a wide variety of foods and plants, are considered to play an important role in the prevention and treatment of osteoporosis. Our previous studies demonstrated that Erythrina cortex extract (EC) rich in prenylated isoflavonoids exerted bone protective effects in ovariectomized (OVX) rats. The present study aimed to investigate the interactions of gut microbiota with the EC extract to explore the underlying mechanisms involved in its beneficial effects on bone. Sprague-Dawley female rats of 3-months-old were ovariectomized and treated with EC extract for 12 weeks. EC extract reversed ovariectomy-induced deterioration of bone mineral density and bone microarchitecture as well as downregulated cathepsin K (*Ctsk*) and upregulated runt-related transcription factor 2 (*Runx2*) and alkaline phosphatase (*ALP*) in the tibia of OVX rats. Its protective effects on bone were correlated with changes in microbial richness and the restorations of several genera. EC increased the serum circulating levels of acetate and propionate in OVX rats. We conclude that the bone protective effects of EC extract were associated with the changes in microbial compositions and serum short chain fatty acids (SCFAs) in OVX rats.

## 1. Introduction

Osteoporosis is a major health problem that affects 200 million people worldwide, and became more and more prevalent due to the increase in the aging population [1]. One in three women over the age of 50 and one in five men of the same age have an osteoporosis-related fracture during their lifetime [2]. Estrogen deficiency was known to be the key factor contributing to the development of osteoporosis; however, recent studies indicated that skeleton metabolism interacted with endocrine, immune, gastrointestinal and renal systems, and was influenced by inflammatory and nutritional status [3,4], especially the gut microbiota (GM) [4]. 

Currently, pharmacologic agents for the treatment of osteoporosis include hormone therapy, bisphosphonates, selective estrogen-receptor modulators, calcitonin, recombinant human parathyroid hormone and monoclonal antibody to receptor activator of nuclear factor κB ligand (RANKL) [1,5,6]. Although these commercially available drugs are effective, most have some limitations and adverse effects that limit their long-term administration and adherence [7,8]. Phytoestrogens are considered to play an important role in hormone deficiency-related symptoms, especially for the prevention and treatment of metabolic syndrome, coronary heart disease and osteoporosis [9,10]. Flavonoids are the major phytoestrogens that are found in a wide variety of fruit, vegetables, grains, flowers, bark, roots, stems, tea and wine [11]. Numerous clinical trials and experimental studies using bone cells and ovariectomized (OVX) animal models have suggested that flavonoids [12,13,14], especially prenylated isoflavonoids [15,16], may serve as an alternative therapy for bone health [17].

Isoflavonoids are reported to be the major phytoconstituents of *Erythrina variegate* L. [18], a folk medicine for managing rheumatic joint pain, spasm of the limbs as well as lower back and knee pain in China, India and Southeast Asia [19]. Our previous studies have clearly demonstrated that the anti-osteoporosis effects of *E. variegate* cortex (EC) extract are mediated by regulating bone turnover and improving bone microstructure and biomechanical strength [20,21]. Subsequently, the major bioactive components in EC were identified to be prenylated isoflavonoids derived from genistein [22,23], which might partially account for the protective effects of EC on bone. Although the protective effects of the prenylated flavonoids-rich EC extract on bone had been demonstrated, the mechanism and systemic actions of the extract involving bone protection in vivo are far from clear.

Recent studies support that the gut microbial community is closely associated with the development and progression of diseases and could affect the body physiology to prevent and combat diseases [24]. Oral administrations of flavonoids were shown to alter the gut microbial ecosystems which led to the changes in the abundance and diversity of the microbial community [25]. Previous studies showed that flavonoids’ aglycones, but not including their glycosides, may inhibit growth of some intestinal bacteria. The bacteria that survived the stress of flavonoids will shape the unique bacterial composition after continuous consumption of special flavonoids by humans [26]. Short-chain fatty acids (SCFAs), the fermented metabolites of non-digestible carbohydrates, are one of the most discussed mechanisms of how gut bacteria impact host physiology [27]. Thus far, there is little knowledge on how prenylated isoflavonoids influence microbial composition and SCFA to prevent osteoporosis.

The current study aims to study the interactions between gut microbiota and the prenylated isoflavonoids-rich EC extract and their potential involvement in meditating the bone protective effects of EC extract.

## 2. Materials and Methods

### 2.1. Herbal Collection and Preparation

The fresh stem bark of *Erythrina variegate* L. (13.0 kg) was collected in January 2016 at Jinghong, Yunnan province, China and identified by Professor Chaozhong Peng from the Institute of Medicinal Plant Development. A voucher specimen (no.SZEC01) was deposited in Shenzhen. The dried stem bark in shade (3.7 kg) was refluxed with 65% ethanol, and the extract was dried to powder (202 g, yield, 5.4%) by a vacuum-drying method.

### 2.2. Prenylated Isoflavonoids’ Identification in EC Extract Using Ultra-Performance Liquid Chromatography Orbitrap-Mass Spectrometry (UPLC-Obitrap-MS) Analysis

A total of 5 mg dried EC extract was re-dissolved in 1 mL methanol with ultra-sonication prior to LC analysis. The LC separation was performed on a Waters ACQUITY UPLC HSS T3 column (2.1 mm × 100 mm, 1.8 μm) with a HSS T3 pre-column (2.1 mm × 5 mm, 1.8 μm) at 40 °C. A gradient elution of solvent A (water with 0.1% formic acid) and solvent B (acetonitrile with 0.1% formic acid) was applied with a modified elution program as follows: 0–10 min, 10–40% B; 10–12 min, 40–55% B; 12–19 min, 55–95% B; 19–22 min, 95% B; 22–25 min, 10% B. The flow rate was 0.3 mL/min, and the injection volume was 3 μL. The sample chamber temperature was 6 °C.

UPLC-Orbitrap-Mass spectrometry analysis was conducted by a Thermo Scientific Orbitrap Fusion Lumos Tribrid mass spectrometer (Thermo Fisher, Waltham, MA, USA) connected to a Waters ACQUITY UPLC System (Waters Corp., CA, USA) with a heated electrospray ionization (H-ESI) interface. The H-ESI-MS spectra were acquired in both positive and negative ion modes. The mass-spectrometric parameters were set as follows: spray voltage, 2300 V and 3500 V in ESI negative and positive ionization modes, respectively (ion transfer tube and vaporizer temperature, 300 °C). Nitrogen gas was used as the sheath gas and the aux gas with a flow rate of 30 arbitrary units and 10 L/min, respectively. The analyzer was operated in a data-dependent acquisition mode, with full MS scans of mass range at 90–1000 mass to charge with detection in the Orbitrap (120,000 resolution) and with auto gain control targeted at 80,000 count and a maximum injection time at 100 ms.

### 2.3. Animal and Treatments

The animal welfare and experimental protocol were performed strictly in accordance with the procedures approved by the Animal Ethic Committee of the Hong Kong Polytechnic University. A total of 48 virgin Sprague-Dawley specific-pathogen-free (SPF) female rats at 3-months-old were purchased from Vital River Laboratory Animal Technology Company (Peking, China). During the study, all rats were housed in the standard condition of 12 h light/12 h dark cycle, 20–22 °C temperature and 40~60% humidity, allowed free access to distilled water and pair-fed the phytoestrogen-free diet (D00031602, Research Diet, NJ, USA), as reported in our previous study [28].

Rats (*n* = 48) were subjected to sham operation (*n* = 12) or bilateral ovariectomy (*n* = 36), and then recovery for 2 weeks. Subsequently, the sham rats were given dd-H_2_O daily, while the OVX rats were divided randomly into three groups and were orally administrated daily for 12 weeks as follows: vehicle-treated (OVX), Premarin-treated (PR, 130 μg/kg) and EC extract-treated (EC, 600 mg/kg). The EC extract was freshly prepared as 60 mg/mL, and the dosage of EC was based on our previous experiment [21]. The dosage of Premarin (Wyeth, PA, USA) was calculated based on the clinical effective dose (1.25 mg conjugated estrogens/day). Before sacrifice, the feces were collected freshly; urine was collected for 24 h; blood was withdrawn from the abdominal aorta, and serum was prepared. The uterus was collected and weighed, and then calculated as the uterus index (UI = uterus weight/body weight). The left tibia and femur were dissected and wrapped in PBS buffer for further analysis.

### 2.4. Chemistries in Serum and Urine

Serum and urine calcium (Ca) were measured by the Arsenazo III colorimetric method using a commercial kit (KHB, Shanghai, China). Serum phosphorous (P) was determined by an inorganic phosphorus kit using the UV method (KHB, Shanghai, China). Serum alkaline phosphatase (ALP) was measured using the AMP buffer method using commercial kits (KHB, Shanghai, China). Urine creatinine was determined by the Sarcosine oxidase-PAP method (KHB, Shanghai, China). All these assays were performed on a Hitachi 7100 automatic analyzer (Hitachi, Japan).

### 2.5. Micro-Computed Tomography (Micro-CT) Analysis of Bone Properties

Bone properties at the proximal metaphysis of the tibia, distal metaphysis of the femur as well as L4 of lumber vertebrae were measured by using Micro-CT (VivaCT 40, Scanco Medical, Brassdrof, Switzerland). The starting scan site was defined as 2.0 mm and 4.0 mm away from the tibia head and femur end, respectively. The bone samples were scanned in the axial direction with a resolution of 21 μm and a scanning power of 70 kVp and 110 μA. One hundred consecutive slices (length of 12.5 μm/slice) were used for contouring the volume of interest (VOI) to evaluate the morphological properties. For lumber vertebrae, a total of 100 continuous slices were scanned in the middle part of L4 (middle point ± 50 slices). The threshold values for contouring VOIs were 200, which were based on the contoured image matched with the grayscale of the background image [29]. Contoured VOI images were evaluated by the Evaluation Program v6.0 (Scanco) to generate 3D bone biological parameters as follows: bone mineral density (BMD), trabecular bone number (Tb.N), trabecular bone separation (Tb.Sp), trabecular bone thickness (Tb.Th), bone volume over total volume (BV/TV), connectivity density (Conn.D) and structure model index (SMI).

### 2.6. Real-Time Quantitative Reverse Transcriptase-Polymerase Chain Reaction (RT-PCR) Analysis

Total RNA was extracted from the right tibia using TRIzol reagent (Invitrogen, Rockville, MD, USA), and transcribed by using a high-capacity cDNA reverse transcription kit (Thermo Scientific, Lithuania, EU). Real-time PCR was performed in the CFX 96 Real Time system (BioRad Laboratories, CA, USA). The PCR program was carried out as follows: denaturation 95 °C for 1 min, amplification for 40 cycles (95 °C for 20 s; 60 °C for 20 s and 72 °C for 18 s). The relative mRNA amount was normalized to GAPDH mRNA, a housekeeping gene. The PCR primers used in this study were listed in Table 1.

### 2.7. Fecal DNA Extraction and PCR Amplification

Fresh fecal samples were collected from each individual rat one day before sacrifice and frozen at −80 °C within 3 h of sampling. DNA extraction was extracted from 0.2 g fecal material, using the Stool Genomic DNA Extraction Kit (Solarbio Life Sciences, Beijing, China). The concentration of bacterial DNA was determined by Qubit Fluorometer (Thermo Scientific, USA).

The V3–V4 region of the bacterial 16S rRNA gene was amplified by PCR with barcode-indexed primers of 515F and 806R using the NEB Phusion High-Fidelity PCR Master Mix. The following PCR primers were used: 515F: 5′-GTGCCAGCMGCCGCGGTAA-3′ and 806R: 5′-GGACTACHVGGGTWTCTAAT-3′. Amplicons were purified with Agencourt AMPure XP beads (Beckman, USA) and were quantified by determining the average molecule length using the Agilent 2100 bioanalyzer instrument and quantifying the library by real-time quantitative PCR. Purified amplicons were pooled in equimolar proportions and paired-end sequenced on the Illumina MiSeq platform with MiSeq Reagent Kit (Illumina, USA). The raw sequencing data were available through the linkage in Appendix A. 

### 2.8. 16S rRNA Sequencing and Bioinformatics Analysis

To determine the composition of gut microbiota in rats belonging to the different groups, operational taxonomic units (OTUs) were clustered with a 97% similarity cut-off by using UPARSE (V.7.0, http://qiime.org/install/index.html, accessed on 4 September 2019), and chimeras were filtered out by using UCHIME (V.4.2). A total of 2,146,617 tags combined by the high-quality paired-end reads were generated, and 756 OUT were clustered. All statistical analyses were performed using R packages (V3.1.1) as in our precious study [30]. The Observed species, Chao index, Ace index, Shannon index and Simpson index were calculated using mothur (version v.1.30.1) to evaluate alpha diversity. Principal components analysis and principal coordinates analysis (PCoA) were performed to evaluate the beta diversity. Taxonomic changes that differed significantly between different groups are shown by pie plot and extended error bar plot. The Spearman’s correlation coefficients were employed to assess bivariate relationships between variables. Results with *p* < 0.05 between groups were considered statistically significant.

### 2.9. Short Chain Fatty Acid (SCFA) Measurements

A total of 50 μL of serum was weighed into a 2 mL polypropylene tube and kept in a cool rack throughout the extraction. A total of 33% HCl (5 μL) was added and mixed with samples, followed by the addition of 1 mL of diethyl ether with mixing for 1 min, and centrifuged for 3 min at 4 °C. The organic phase was transferred into a 2 mL GC vial.

For the calibration curve, 100 μL of SCFA calibration standards (Sigma) were dissolved in water to concentrations of 0, 0.25, 0.5, 1, 1.5 and 2.5 mM and then subjected to the same extraction procedure as the samples.

For GC analysis, 1 μL of the sample was injected with a split ratio of 10:1 on a ZB-WAX Plus, 30 m × 0.25 mm iD, 0.25 μm df capillary column (Phenomenex, USA) and analyzed by the Agilent GC7890B gas chromatograph (Agilent, USA). Helium was used as the carrier gas with a constant flow rate of 1 mL/min. The injection temperature was 200 °C. The column temperature program started with 80 °C and was ramped to 210 °C at a rate of 20 °C/min and then held at 210 °C for 2 min. SCFAs were identified based on the retention time of standard compounds. Quantification was done by integration of the peak area for the following retention times: acetate eluted at 3.54 min, propionate eluted at 3.96 min and butyrate eluted at 4.42 min.

### 2.10. Statistical Analysis

The pharmacodynamic data were presented as mean ± SEM. Differences were analyzed statistically with one-way analysis of variance (ANOVA) followed by Tukey’s post-hoc test using Graphpad PRISM software package. *p* < 0.05 was considered statistically significant.

## 3. Results

### 3.1. The Prenylated Isoflavonoids Identified in EC Extract

A total of 63 flavonoids isolated from *Erythrina variegate* L. were obtained by literature research and listed in Supplementary Table S1. The major flavonoids in EC extract were identified by UPLC-Orbitrap-MS analysis based on the fragmentation information of each peaks, due to unavailable commercial standards of these flavonoids. Totally, 29 prenylated flavonoids were identified in EC extract and are shown in Supplementary Table S2.

### 3.2. EC Exerted Protective Effects on Ovariectomy-Induced Bone Loss

The bone protective effects of EC extract were evaluated in OVX rats. Ovariectomy significantly increased the body weight, altered serum and urine Ca as well as serum alkaline phosphatase (ALP) in rats (Table 2). Treatment with Premarin (PR) did not alter body weight but significantly restored serum Ca and ALP in OVX rats. Treatment with EC did not alter body weight but could significantly decrease serum ALP level and suppressed urine Ca in OVX rats, and the results were consistent with our previous study [21]. Estrogen deficiency induced serious bone loss and bone microstructure deterioration at the tibia, femur and lumbar vertebra in OVX rats as revealed by micro-CT analysis. As shown in Table 2, ovariectomy significantly decreased bone mineral density (BMD), trabecular bone number (Tb.N), trabecular bone thickness (Tb.Th), trabecular bone fraction (BV/TV) and connective density (Conn.D) while it increased trabecular bone separation (Tb.Sp) and the structure model index (SMI) at the proximal metaphysis of the tibia, distal metaphysis of the femur and the L4 of lumbar vertebra in OVX rats (vs. Sham rats). Treatment with PR and EC significantly reversed the deterioration induced by ovariectomy at the tibia, and improved several microarchitectural parameters at the femur and lumber vertebrate in OVX rats. Unlike PR, EC did not increase uterus weight in OVX rats.

### 3.3. Effects of EC Treatment on Regulating Osteoblast-Specific and Osteoclast-Specific mRNA Expressions

**Figure 1:**
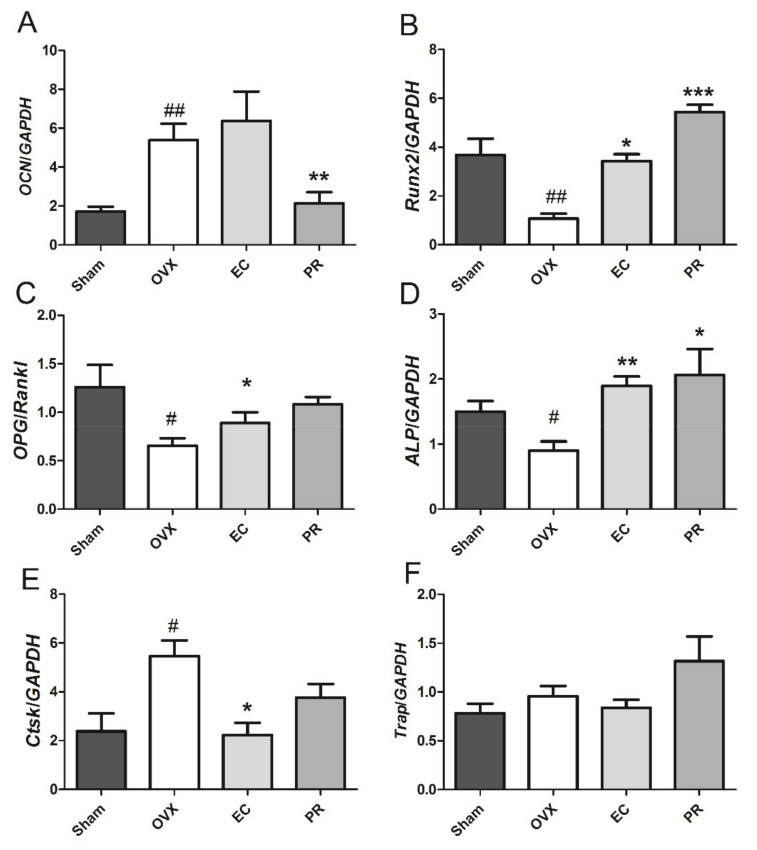
Effects of Erythrinae Cortex (EC) extract on bone specific mRNA expression in rat tibia. Three-month old SD rats were subjected to the following treatment for 12 weeks after ovariectomy: Sham, Sham-operated, vehicle-treated; OVX, ovariectomized, vehicle-treated; PR, ovariectomized, Premarin-treated (0.13 mg/kg); EC, ovariectomized, EC extract-treated (600 mg/kg). (**A**) *OCN*, osteocalcin; (**B**) *Runx2*, runt-related transcription factor 2; (**C**) *OPG*/*Rankl*, *OPG*, osteoprotegerin; *Rankl*, receptor activator of nuclear factor κB ligand; (**D**) *ALP*, alkaline phosphatase; (**E**) *Ctsk*, cathepsin K; (**F**) *Trap*, tartrate-resistant acid phosphatase 5; *GAPDH*, glyceraldehyde 3-phosphate dehydrogenase. Results were expressed as mean ± SEM (*n* = 6). # *p* < 0.05, ## *p* < 0.01 vs. Sham; * *p* < 0.05, ** *p* < 0.01, *** *p* < 0.001 vs. OVX.

To investigate the mechanism of the actions of EC on bone remodeling further, the mRNA expressions of osteoblast-specific genes, namely osteocalcin (*OCN*), *ALP*, runt-related transcription factor 2 (*Runx2*) and the ratio of osteoprotegerin and receptor activator of nuclear factor κB ligand (*OPG/Rankl*), and osteoclast-specific genes, namely cathepsin k (*Ctsk*) and tartrate resistant acid phosphatase (*Trap*), were measured. As shown in Figure 1, the mRNA expressions of *Runx2*, *ALP* and *OPG*/*Rankl* were decreased, while *OCN* and *Ctsk* were increased in rat tibia in response to ovariectomy. The mRNA expressions of *ALP* and *Runx2* were significantly upregulated in both PR- and EC-treated rats. Most importantly, the mRNA expression of *Ctsk* was dramatically suppressed in OVX rats in response to EC treatment, but not to PR treatment. The mRNA expressions of *OCN* were downregulated, and *OPG*/*Rankl* was upregulated in OVX rats by PR treatment, but not by EC treatment. These results indicated that the beneficial effects of EC on bone were mediated by modulating the expression of genes involved in both osteoblastogenesis and osteoclastogenesis.

### 3.4. Effects of EC Extract on the Gut Microbiota Structure in Ovariectomized Rats

**Figure 2:**
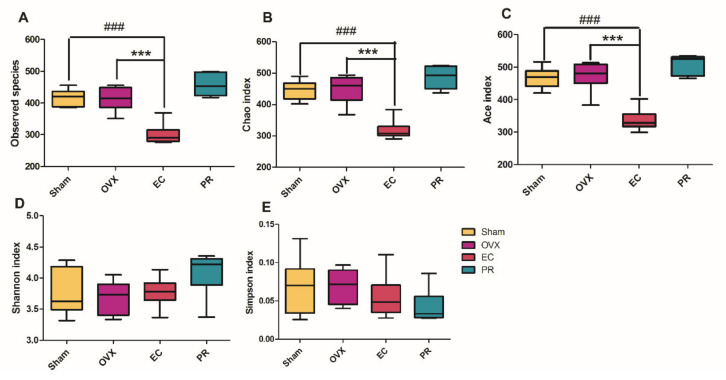
Changes in fecal microbial diversities upon different treatments measured by α-diversity on observed operational taxonomic unit (OTU). (**A**) Observed species; (**B**) Chao index; (**C**) Ace index; (**D**) Shannon index; (**E**) Simpson index. Statistical analysis was performed by one-way analysis of variance (ANOVA) followed by Tukey’s post-hoc test (*n* = 6), and ### *p* < 0.001 vs. Sham; *** *p* < 0.001 vs. OVX.

To investigate the influence of the prenylated isoflavonoid-rich EC extract on gut microbiota in OVX rats, we analyzed the composition of the gut microbial community by 16S rRNA gene pyrosequencing. The number of operational taxonomic unites (OTUs) and taxa at different levels was almost consistent in vehicle-treated OVX rats, Sham rats and PR treatment rats, while OTU and taxa at all levels tended to decrease in EC-treated OVX rat, as shown in Table 3. As shown in Figure 2, ovariectomy did not influence the species’ richness of the gut microbiota as indicated by the Sobs index, Chao index and Ace index, as well as the species diversity as measured by the Shannon index and Simpson index (OVX vs. Sham rats). EC treatment significantly reduced the Sobs index, Chao index and Ace index, but did not alter the microbiota diversity in OVX rats as compared to those of Sham and vehicle-treated OVX rats. The results indicated that estrogen deficiency or supplement is not a key factor for maintaining or altering the gut microbial composition in our current animal condition. It also suggested that the abundance of special genera, but not gut microbiota diversity, were altered in response to EC treatment in mature rats.

**Figure 3:**
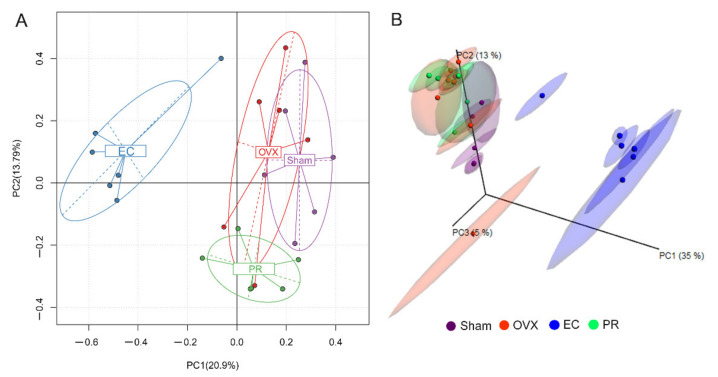
PCA and PCoA plots based on operational taxonomic unit (OTU) abundance. (**A**) Principal component analysis (PCA) plot. (**B**) Principal coordinate analysis (PCoA) plot of the unweighted UniFrac distance matrix. Number in brackets represents contributions of principal components (**A**) or coordinate (**B**) to differences among samples. A dot represents each sample, and different colors represent different groups.

The impact of EC extract on the gut microbial structure was further assessed by performing principal component analysis (PCA) and principal coordinate analysis (PCoA). The PCA analysis based on 97% operational taxonomic unit (OTU) abundance matrix of microbiota revealed an extremely distinct separation between the EC treated group and the other three groups (Figure 3A). In Figure 3B, unweighted UniFrac analysis also showed that PCoA could discriminate the EC treated group from the other groups. In contrast, there was substantial overlap in both PCA and PCoA plots among the Sham, OVX and PR group, suggesting that chronic estrogen deficiency (14 weeks post-ovariectomy) or estrogen supplement did not result in significant alteration of the microbial community. The results also indicate that EC treatment appeared to induce substantial changes in the microbial community.

**Figure 4:**
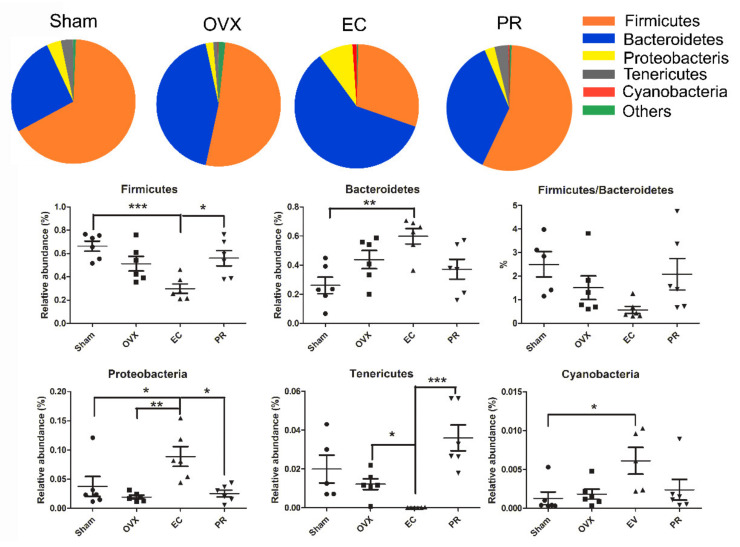
Community pie-plot analysis and microbiota alternation at Phylum level in Sham, OVX, EC and PR-treated OVX rats. *n* = 6 in each group. The dashes in the scalar diagram indicate means ± SEM. * *p* < 0.05, ** *p* < 0.01, *** *p* < 0.001.

A taxon-based analysis also revealed marked changes in gut microbial compositions at phylum and genus levels in response to EC treatment. In Figure 4, the bacterial taxa of OVX rats did not exhibit significant differences with that of Sham rats. PR treatment showed a similar phylum and genus composition with the Sham and OVX group. As compared to Sham and vehicle treated OVX rats, the EC extract significantly increased the abundance of *Bacteroidetes, Proteobacteria*, *Cyanobacteria* and reduced the abundance of *Firmicutes* in OVX rats. The results further indicated that estrogen seems not a key factor for influencing gut microbial composition.

**Figure 5:**
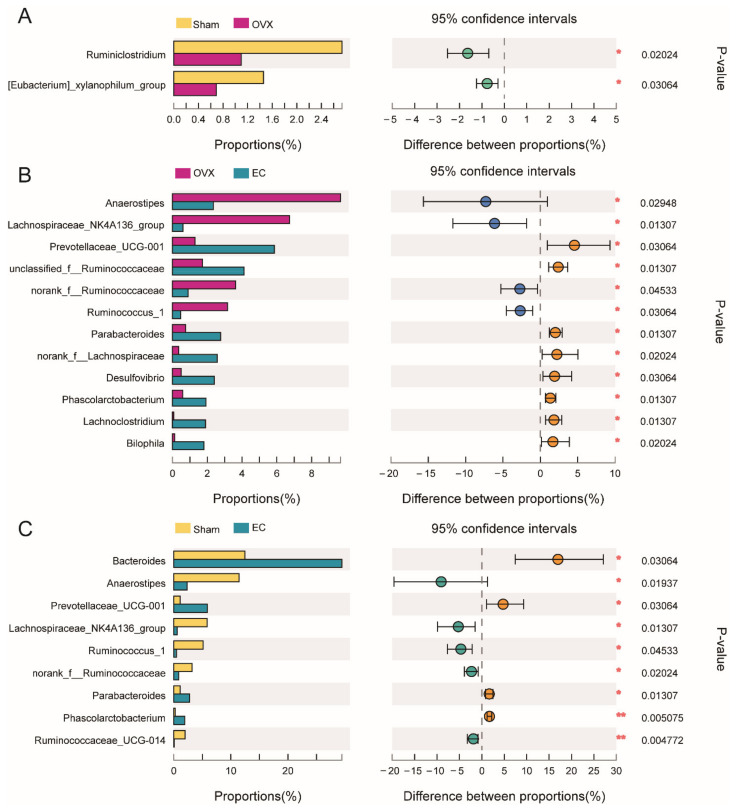
Extended error bar plot identifying the significant differences of phylotypes between mean proportions of top 20 bacterial taxa at genus level. (**A**) Sham vs. OVX; (**B**) OVX vs. EC; (**C**) Sham vs. OVX. Data were showed as relative abundance (%). *n* = 6 in each group. Statistical analysis was performed by the Wilcoxon rank-sum test, and * *p* < 0.05, ** *p* < 0.01.

As shown in Figure 5, only two characteristic microbes of the top 20 taxa at the genus level, *Ruminiclostridium* and *(Eubacterium)_xylanophilum_group*, were highly suppressed in rats by ovariectomy (vs. Sham rats). This finding further indicated that chronic estrogen deficiency did not result in great changes in total gut microbial composition. Meanwhile, EC treatment was characterized by the high abundance of *norank_f_Lachnospiraceae*, *Desulfovibrio*, *Lachnoclostridium* and *Biophila* in OVX rats (vs. vehicle-treated OVX rats). Furthermore, these altered genera upon EC treatment compared with OVX rats were different from that compared with Sham rats in the top 20 genera.

**Figure 6:**
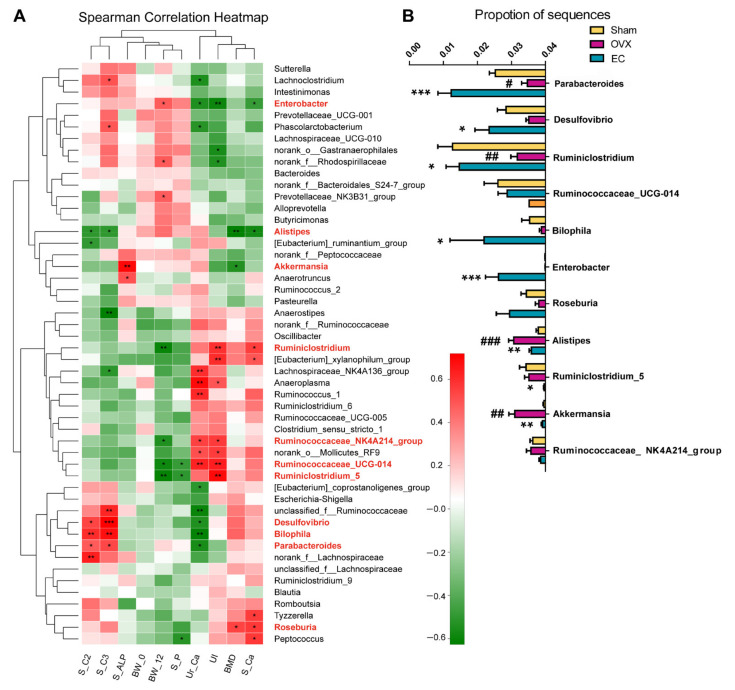
The correlation between relative abundance (%) of top 50 bacterial taxa and bone related indicators at genus level. (**A**) correlation heatmap; (**B**) the changes of 5 genera that closely correlated with bone indicators in Sham, OVX and EC-treated OVX rat microbiotas. The correlation coefficient was determined by the Spearman index. Red, positive correlation; Green, negative correlation. * *p* < 0.05, ** *p* < 0.01 *** *p* < 0.001 for (**A**). # *p* < 0.05, ## *p* < 0.01, ### *p* < 0.001 vs. Sham; * *p* < 0.05, ** *p* < 0.01, *** *p* < 0.001 vs. OVX for (**B**).

To evaluate the potential relationships between EC-induced changes in the top 50 gut microbiota composition at the genus level and several indicators (BMD, UI, S_Ca, S_P, Ur_Ca, S_ALP, BW_0, BW_12), the Spearman’s correlation analysis was performed (Figure 6A). The results showed that serum calcium (S_Ca), body weight at 12 weeks (BW_12), uterus index (UI) and urine calcium (Ur_Ca) were significantly correlated with more than six genera, while bone mineral density (BMD), serum phosphorous (S_P), serum alkaline phosphatase (S_ALP) showed correlations with fewer than or equal to three genera in mature rats. As expected, body weight at 0 weeks (BW_0) did not exhibit any relationship with gut microbiota in mature rats. *Enterobacter*, *Alistipes*, *Akkermansia*, *Ruminiclostridium*, *Ruminococcaceae_NK4A214_group*, *Ruminococcaceae_UCG-014*, *Ruminiclostridium_5* and *Roseburia* were genera found to be correlated significantly with bone indicators. As shown in Figure 6B, EC treatment significantly restored the level of *Ruminiclostridium* and suppressed the level of *Alistipes* and *Akkermansia* induced by ovariectomy in rats. In addition, it dramatically increased the abundance of *Enterobacter* and decreased the abundance of *Ruminiclostridium_5* in OVX rats (vs. Sham and vehicle treated OVX rats). The above results indicated that the composition of gut microbiota and the abundance of several special genera appeared to associate with bone indicators and might account for the bone anabolic effects of EC extract.

### 3.5. The Contents of SCFA in Serum and Its Actions on Bone

**Figure 7:**
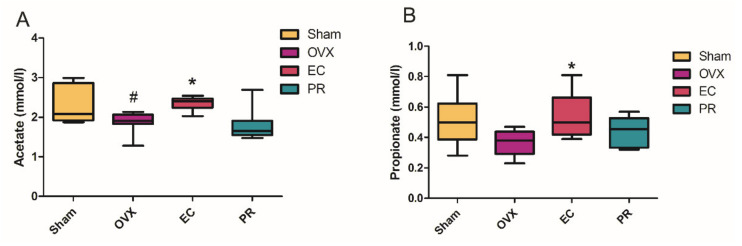
The effects of Erythrinae Cortex (EC) extract treatment on the serum levels of short chain fatty acids (SCFAs). (**A**) the levels of acetate; (**B**) the levels of propionate. Values represent the mean SEM. Statistical analysis was performed by one-way analysis of variance (ANOVA) followed by Tukey’s post-hoc test (*n* = 10–12). # *p* < 0.05 vs. Sham and * *p* < 0.05 vs. OVX.

The short chain fatty acids (SCFA), the major end metabolites of carbohydrates in the gut, play an important role in bone homeostasis. Serum concentrations of major SCFAs (acetate, propionate and butyrate) in rats in response to ovariectomy and treatment with EC were determined using the GC-FID method. As shown in Figure 7, the serum level of acetate was significantly decreased in OVX rats (vs. Sham rats), and a propionate showed a decreased trend in OVX rats compared with Sham rats. Both of acetate and propionate were significantly increased in rats treated with EC (vs. OVX rats), while PR treatment did not alter the levels of these two SCFAs. The serum butyrate level was undetectable in all samples.

Spearman’s correlation analysis indicated that serum acetate (S_C2) and propionate (S_C3) in rats were positively correlated with the abundance of *Desulfovibrio*, *Biophila* and *Parabacteroides* in gut microbiota (Figure 6A). These bacterial taxa were also found to correlate negatively with urine Ca in rats. In addition, the abundance of *Alistipes* was found to correlate negatively with serum acetate, propionate, Ca and BMD in rats. In Figure 6B, EC treatment significantly increased the abundance of *Parabacteroides*, *Desulfovibrio* and *Biophila*, and decreased the abundance of *Alistipes* in OVX rats (vs. vehicle treated OVX rats). These results suggested that serum acetate and propionate might serve as novel bone markers that reflect the influence of treatment on gut microbiota.

## 4. Discussion

We aim to investigate if the anabolic effects of EC extraction on bone is associated with the change in composition and metabolites of gut microbiota. The novel findings of the present study include: (1) the protective effects of EC extract on bone was correlated to the changes of the microbial richness and the restorations of several specific microbial genera (Figure 1, Figure 2, Figure 3, Figure 4, Figure 5 and Figure 6 and Table 1, Table 2 and Table 3); (2) EC treatment increased short chain fatty acids concentrations in serum, which was correlated with several microbial genera (Figure 6 and Figure 7).

Li et al. [31] reported that sex steroid-depleted mice in a germ-free condition were protected from trabecular bone loss, and the capacity of sex steroid deficiency to induce bone loss was restored after microbial recolonization, which indicated that bone loss due to estrogen deficiency is gut microbiota-dependent. In addition, several probiotics, such as *Lactobacillus rhamnosus GG*, *Lactobacillus aciphilus*, *Lactobacillus casei* and *Lactobacillus reuteri 6475*, showed protective effects against bone loss in mice induced by ovariectomy [31,32]. The latter further suggested that intestinal microbiota has a close relationship with bone metabolism. Several studies investigated how gut microbiota changed in patients or animal models with osteoporosis. Wang et al. firstly revealed that BMD was in an inverse correlation with the number of bacterial taxa in the fecal samples of osteoporosis and osteopenia patens compared with those in the control group [33]. In our current study, the number of OTU and taxa at each levels was consistent in Sham and OVX rats, while the number was significantly decreased in EC-treated OVX rats (Table 3). Estrogen seems not a critical factor for gut microbiota alternation in our study. 

The inhibitory effects of EC extract to the bacterial taxa can be easily explained by the known antibacterial activities of many isoflavonoids [16]. The populations of *Bateriodetes* (B) and *Firmicutes* (F) in OVX rats showed a decreasing trend without any statistical significance compared with the Sham group which was in accordance with the decrease in F:B in both OVX rats and mice in publications [34,35]. However, recently, a clinical study reported opposite results that the low-BMD individuals had a smaller number of bacterial taxa, and F:B was positively correlated with the BMD in OP patients [36]. All the above studies suggested that gut microbiota is a key regulator of bone mass, but the interactions between estrogen deficiency-induced bone loss and the microbiota are far from being clearly understood with the limited studies.

In our current study, BMD, S_Ca, S_P, S_ALP, U_Ca and body weight before (BW_0)/after (BW_12) treatment were selected as the bone metabolic indicators to analyze the correlation with gut microbiota. Interestingly, although BMD is a widely used parameter for diagnosing osteoporosis, it exerted fewer correlations with bacterial taxa compared with other indicators. The reason might be that biochemical markers could offer a dynamic and global analysis of the skeleton which can correlate with the dynamic microbiota better, while BMD is a relative static parameter [37]. In addition, our results (Figure 6) showed that *Ruminiclostridium*, *Roseburia*, *Alistipes*, *Akkermansia* and *Enterobacter* are correlated with bone metabolic indicators, and EC treatment significantly restored the changes of these genus induced by ovariectomy. Our results is accordance with a recent study on *Roseburia* which reported its positive correlation with BMD and T-score [36]. The above results indicated that microbiota indeed relates to bone metabolism and played an important role in mediating the protective effects of EC on bone.

Short chain fatty acids (SCFAs), a kind of saturated aliphatic organic acids with a backbone of one to six carbons, have caught worldwide attention for their significant physiological and pharmacological effects. They are primarily produced in the large intestine via fermentation of dietary fibers and are resistant starches by gut microbiota. Acetate, propionate and butyrate are the most abundant SCFAs in the gastrointestinal tract (≥95%) with a molar ratio of 3:1:1 [38]. Several factors including strain and quantity of gut microbiota, substrate source, host genotype and intestinal transit time influence the release of SCFAs. Several studies reported that SCFAs could influence bone homeostasis by activating the immune system and directly inhibiting osteoclast activities [39,40]. SCFAs act as a link between the microbiota and immune system by affecting the activation and effector function of T cells which intimately correlated to bone homeostasis [39,41]. Lucas et al. firstly reported that the bone protective effects of propionate and butyrate were directly related to the inhibition of osteoclast differentiation and bone resorption in vivo and in vitro [42]. Jia et al. discovered that berberine could protect against estrogen deficiency-associated alveolar bone loss by upregulating butyrate generation by gut microbiota, restoring the intestinal barrier and inhibiting immune abnormality [43]. The concentrations of SCFAs in feces obtained from the present animal study were determined, and the results showed that the SCFAs levels were similar in all treatment groups (data are not shown), indicating that EC treatment did not alter the levels of SCFAs in rat feces. In contrast, serum concentrations of acetate and propionate were significantly increased in OVX rats upon EC treatment (vs. vehicle treated OVX rats) (Figure 7). The seemingly unexplainable results might be due to that fecal SCFAs cannot reflect the actual amount of intestinal SCFAs which are mainly produced in cecum [44]. In addition, about 95% SCFAs produced by gut fermentation were quickly absorbed from mucosa, while only 5% of them were excreted daily in the feces [45]. Van Beer-Schreurs et al. reported that the concentration of SCFAs remained unchanged in the intestinal lumen of piglets, when the SCFAs’ concentrations in portal blood increased after giving a solid diet during weaning. These data suggest that the concentration of luminal and fecal SCFAs do not always reflect the concentrations of absorbed SCFAs, which is inconsistent with our study. Absorbed SCFAs may play a more important role in physiology.

Moreover, subsequent Spearman correlation analysis demonstrated that serum acetate and propionate correlated with the changes of several genera, such as *Parabacteroides*, *Desulfovibrio*, *Biolophila* and *Alistipes*, which were also shown to correlate with BMD and bone metabolic markers. *Parabacteroides* and *Alistipes* were reported to be SCFA-producing bacteria [46,47]. The abundance of *Parabacteroides* was enriched upon EC treatment, while the abundance of *Alistipes* was decreased. The levels of acetate and propionate were increased in the EC treated group. The results suggested that the SCFAs levels may depend on the balance of different bacteria. Our results could suggest that EC improve bone properties through the alteration of the abundance of some bacteria which subsequently lead to the alteration of serum levels of SCFAs. The limitation of the present study is that it could not provide direct mechanistic evidence for the involvement of microbiome/SCFA in mediating the actions of EC on bone. Further study will be needed to use antibiotics to test if EC require the microbiome for exerting its actions.

## 5. Conclusions

The present study revealed that the bone anabolic effects of the prenylated isoflavonoids-rich extract (EC) from Erythrinae Cortex was directly correlated with the restorations of changes in several microbial genera induced by ovariectomy. The results also demonstrated that the diversity of gut microbiota was not changed by ovariectomy nor treatment, while the microbial richness might serve as a biomarker for monitoring bone status under estrogen deficiency. In addition, our study indicated that SCFAs might also be involved in mediating the bone protective actions of EC extract, as the increase in serum SCFAs in rats upon EC treatment was associated with the changes in several gut bacterial taxa that were also correlated with the changes in BMD and urinary Ca excretion in rats. Taken together, the bone protective effects of EC extract are partially mediated by the changes in microbial richness and the increase in circulating SCFAs. Our study provides insight for understanding the unique mechanism by which prenylated isoflavonoids-rich extract interacts with gut microbiota to exert bone protective effects in vivo.

## Figures and Tables

**Table 1:** Primers used for Real-time RT-PCR.

Primer	Accession No.	Sequence (5′-3′)	Tm (°C)
*Runx2*	NM_001278484	F: TAACGGTCTTCACAAATCCTC	56
		R: GGCGGTCCAGAGAACAAACTA	
*ALP*	NM_013059	F: GTGGTGGACGGTGAACGGGAGAA	53
		R: ATGGACGCCGTGAAGCAGGTGAG	
*OCN*	NM_013414	F: GCAGCTTCAGCTTTGGCTACTCT	56
		R: CAACCGTTCCTCATCTGGACTTTA	
*OPG*	NM_012870	F: ACAATGAACAAGTGGCTGTGCTG	55
		R: CGGTTTCTGGGTCATAATGCAAG	
*Rankl*	NM_057149	F: GCAGCATCGCTCTGTTCCTGTA	55
		R: GCATGAGTCAGGTAGTGCTTCTGTG	
*Ctsk*	NM_031560	F: CGCCACGGCAAAGGCAGCTAA	64
		R: CGGGTCCTACCCGAGCCACT	
*Trap*	NM_001270889	F: ACCGCCTACCTGTGTGGGCA	62
		R: CCATGAAGTTGCCGGCCCCA	
*GAPDH*	NM_017008	F: CAAGTTCAACGGCACAGTCAAGG	51
		R: ACATACTCAGCACCAGCATCACC	

*Runx2*, runt-related transcription factor 2; *ALP*, alkaline phosphatase; *OCN*, osteocalcin; *OPG*, osteoprotegerin; *Rankl*, receptor activator of nuclear factor κB ligand; *Ctsk*, Cathepsin K; *Trap*: tartrate resistant acid phosphatase 5; *GAPDH*, glyceraldehyde 3-phosphate dehydrogenase.

**Table 2:** Bone parameters, body weight changes, uterine index and levels of biochemical markers in serum and urine of rats.

		Sham	OVX	EC	PR
Tibia	BMD (mgHA/cm^3^)	360.0 ± 11.8	118.3 ± 6.3 ###	179.5 ± 17.1 *	293.2 ± 14.2 ***
	Tb.N (mm^−1^)	3.93 ± 0.20	1.18 ± 0.02 ###	1.67 ± 0.21	3.11 ± 0.19 ***
	Tb.Sp (mm)	0.22 ± 0.01	0.87 ± 0.02 ###	0.68 ± 0.07 *	0.31 ± 0.02 ***
	Tb.Th (mm)	0.133 ± 0.004	0.099 ± 0.002 ###	0.106 ± 0.001	0.120 ± 0.003 *
	BV/TV (%)	0.42 ± 0.02	0.08 ± 0.01 ###	0.15 ± 0.02 *	0.31 ± 0.02 ***
	Conn. D (mm^−3^)	60.9 ± 3.6	8.6 ± 0.8 ###	22.6 ± 4.4 *	45.7 ± 3.9 ***
	SMI	0.54 ± 0.22	2.47 ± 0.07 ###	1.99 ± 0.08 **	1.28 ± 0.17 ***
Femur	BMD (mgHA/cm^3^)	341.6 ± 15.9	112.7 ± 5.5 ###	164.5 ± 10.0 *	265.9 ± 16.7 ***
	Tb.N (mm^−1^)	3.28 ± 0.33	1.31 ± 0.06 ###	1.46 ± 0.12	2.23 ± 0.17 *
	Tb.Sp (mm)	0.33 ± 0.05	0.82 ± 0.05 ###	0.73 ± 0.07	0.51 ± 0.05 **
	Tb.Th (mm)	0.147 ± 0.005	0.105 ± 0.003 ###	0.111 ± 0.002	0.123 ± 0.003
	BV/TV (%)	0.40 ± 0.03	0.07 ± 0.01 ###	0.15 ± 0.01 *	0.28 ± 0.03 ***
	Conn. D (mm^−3^)	44.7 ± 3.7	9.0 ± 1.3 ###	21.4 ± 3.2 *	40.2 ± 3.5 ***
	SMI	0.29 ± 0.28	2.40 ± 0.07 ###	1.72 ± 0.10	0.93 ± 0.23 ***
Lumbar Vertabra	BMD (mgHA/cm^3^)	385.1 ± 12.9	214.2 ± 9.6 ###	271.5 ± 15.8 *	338.3 ± 13.8 ***
	Tb.N (mm^−1^)	3.61 ± 0.14	2.25 ± 0.14 ###	2.82 ± 0.19	3.33 ± 0.10 ***
	Tb.Sp (mm)	0.24 ± 0.02	0.44 ± 0.03 ###	0.34 ± 0.03 *	0.27 ± 0.01 ***
	Tb.Th (mm)	0.14 ± 0.02	0.10 ± 0.01	0.11 ± 0.02	0.11 ± 0.02
	BV/TV (%)	0.46 ± 0.02	0.17 ± 0.01 ###	0.27 ± 0.03 *	0.39 ± 0.02 ***
	Conn. D (mm^−3^)	34.1 ± 2.33	17.6 ± 1.89 ###	30.3 ± 3.8 *	33.9 ± 1.38 ***
Weight change (%)		16.2 ± 1.6	27.6 ± 2.1 ###	26.2 ± 1.7	22.5 ± 1.8
Uterus index (UI, mg/g)		1.87 ± 0.07	0.33 ± 0.02 ###	0.30 ± 0.01	0.91 ± 0.06 ***
Serum Ca (mmol/L)		2.71 ± 0.02	2.50 ± 0.01 ###	2.53 ± 0.02	2.59 ± 0.03 *
Serum P (mmol/L)		1.10 ± 0.04	1.30 ± 0.04	1.29 ± 0.06	1.18 ± 0.08
Serum ALP (ng/mL)		47.7 ± 6.3	107.6 ± 12.7 ###	74.6 ± 3.5 *	70.3 ± 6.0 *
Urine Ca/Cr		0.071 ± 0.010	0.158 ± 0.048	0.029 ± 0.005 **	0.078 ± 0.025

Three-month old SD rats were subjected to the following treatment for 12 weeks after ovariectomy: Sham, Sham-operated, vehicle-treated; OVX, ovariectomized, vehicle-treated; PR, ovariectomized, Premarin-treated (0.13 mg/kg); EC, ovariectomized, EC extract-treated (600 mg/kg). Results were expressed as mean ± SEM. ### *p* < 0.001 versus Sham; * *p* < 0.05, ** *p* < 0.01, *** *p* < 0.001 versus OVX.

**Table 3:** Bacterial operational taxonomic unites and taxa in each group at different levels.

	Sham	OVX	EC	PR
OTU	722	739	565	756
Phylum	18	17	14	18
Class	31	29	24	30
Order	51	48	36	47
Family	80	80	62	75
Genus	174	175	147	166
Species	295	302	252	300

## Data Availability

The data presented in this study are available on request from the corresponding author.

## Supplementary Materials

The following are available online at https://www.mdpi.com/article/10.3390/nu13092943/s1, Table S1: The summary of the isolated flavonoids from *Erythrina variegate* L. obtained by literature research, Table S2: Identified prenylated isoflavonoids from EC extract by ESI-Orbitrap-MS analysis.

## Appendix A

The raw sequencing data for this article are available at http://www.ncbi.nlm.nih.gov/bioproject/743774 upon publication.

## References

[1] Cotts K.G., Cifu A.S. (2018). Treatment of osteoporosis. JAMA.

[2] Carolyn B. (2017). Osteoporosis: Staying strong. Nature.

[3] Stavre Z., Upchurch K., Kay J., Gravallese E. (2016). Differential effects of inflammation on bone and response to biologics in rheumatoid arthritis and spondyloarthritis. Curr. Rheumatol. Rep..

[4] Gérard K., Mathieu F. (2012). The contribution of bone to whole-organism physiology. Nature.

[5] Solomon C.G., Black D.M., Rosen C.J. (2016). Postmenopausal osteoporosis. NEJM.

[6] Watts N.B., Manson J.E. (2017). Osteoporosis and fracture risk evaluation and management: Shared decision making in clinical practice. JAMA.

[7] Sambrook P., Cooper C. (2006). Osteoporosis. Lancet.

[8] Rachner T.D., Khosla S., Hofbauer L.C. (2011). Osteoporosis: Now and the future. Lancet.

[9] Fu S.-W., Zeng G.-F., Zong S.-H., Zhang Z.-Y., Zou B., Fang Y., Lu L., Xiao D.-Q. (2014). Systematic review and meta-analysis of the bone protective effect of phytoestrogens on osteoporosis in ovariectomized rats. Nutr. Res..

[10] Chiang S.-S., Pan T.-M. (2013). Beneficial effects of phytoestrogens and their metabolites produced by intestinal microflora on bone health. Appl. Microbiol. Biotechnol..

[11] Kim K., Kim K., Vance T.M., Vance T.M., Chun O.K., Chun O.K. (2016). Estimated intake and major food sources of flavonoids among US adults: Changes between 1999–2002 and 2007–2010 in NHANES. Eur. J. Nutr..

[12] Tai T., Tsai K., Tu S., Wu J., Chang C., Chen C., Shaw N., Peng H., Wang S., Wu C. (2012). The effect of soy isoflavone on bone mineral density in postmenopausal Taiwanese women with bone loss: A 2-year randomized double-blind placebo-controlled study. Osteoporos. Int..

[13] Nash L.A., Ward W.E. (2017). Tea and bone health: Findings from human studies, potential mechanisms, and identification of knowledge gaps. Crit. Rev. Food Sci. Nutr..

[14] Wang Z., Wang D., Yang D., Zhen W., Zhang J., Peng S. (2018). The effect of icariin on bone metabolism and its potential clinical application. Osteoporos. Int..

[15] Ming L.-G., Lv X., Ma X.-N., Ge B.-F., Zhen P., Song P., Zhou J., Ma H.-P., Xian C.J., Chen K.-M. (2013). The prenyl group contributes to activities of phytoestrogen 8-prenynaringenin in enhancing bone formation and inhibiting bone resorption in vitro. Endocrinology.

[16] Chen X., Emmanuel M., Wong M.-S., Zhang Y. (2014). A systematic review on biological activities of prenylated flavonoids. Pharm. Biol..

[17] Gómez-Zorita S., González-Arceo M., Fernández-Quintela A., Eseberri I., Trepiana J., Portillo M.P. (2020). Scientific evidence supporting the beneficial effects of isoflavones on human health. Nutrients.

[18] Kumar A., Lingadurai S., Jain A., Barman N.R. (2010). Erythrina variegata Linn: A review on morphology, phytochemistry, and pharmacological aspects. Pharmacogn. Rev..

[19] Song L.R., Song L.R., Hong X., Ding X.L., Zang Z.Y. (2001). Dictionary of Modern Medicine.

[20] Zhang Y., Li Q., Li X., Wan H.Y., Wong M.-S. (2010). Erythrina variegata extract exerts osteoprotective effects by suppression of the process of bone resorption. Br. J. Nutr..

[21] Zhang Y., Li X.L., Lai W.P., Chen B., Chow H.K., Wu C.F., Wang N.L., Yao X.S., Wong M.S. (2007). Anti-osteoporotic effect of Erythrina variegata L. in ovariectomized rats. J. Ethnopharmacol..

[22] Li X., Wang N., Wong Man S., Albert S.C.C., Yao X. (2006). Four new isoflavonoids from the stem bark of Erythrina variegata. Chem. Pharm. Bull..

[23] Zhang Y., Li X.-L., Yao X.-S., Wong M.-S. (2008). Osteogenic activities of genistein derivatives were influenced by the presence of prenyl group at ring a. Arch. Pharm. Res..

[24] Wallace T.C., Guarner F., Madsen K., Cabana M.D., Gibson G., Hentges E., Sanders M.E. (2011). Human gut microbiota and its relationship to health and disease. Nutr. Rev..

[25] Lyu M., Wang Y.-f., Fan G.-w., Wang X.-y., Xu S.-y., Zhu Y. (2017). Balancing herbal medicine and functional food for prevention and treatment of cardiometabolic diseases through modulating gut microbiota. Front. Microbiol..

[26] Huang J., Chen L., Xue B., Liu Q., Ou S., Wang Y., Peng X. (2016). Different flavonoids can shape unique gut microbiota profile in vitro. J. Food. Sci..

[27] Woting A., Blaut M. (2016). The intestinal microbiota in metabolic disease. Nutrients.

[28] Xiao H.H., Dai Y., Wan H.Y., Wong M.S., Yao X.S. (2011). Bone-protective effects of bioactive fractions and ingredients in *Sambucus williamsii* HANCE. Br. J. Nutr..

[29] Wong K.C., Shing L.K., Kit L.H., Ying W.H., Kwan H.C., Zhang Y., Sau W.M. (2014). Er-Xian decoction exerts estrogen-like osteoprotective effects in vivo and in vitro. Am. J. Chin. Med..

[30] Xiao H.-H., Lu L., Poon C.C.-W., Chan C.-O., Wang L.-J., Zhu Y.-X., Zhou L.-P., Cao S., Yu W.-X., Wong K.Y. (2021). The lignan-rich fraction from Sambucus Williamsii Hance ameliorates dyslipidemia and insulin resistance and modulates gut microbiota composition in ovariectomized rats. Biomed. Pharmacother..

[31] Li J., Chassaing B., Tyagi A., Vaccaro C., Luo T., Adams J., Darby T., Weitzmann M., Mulle J.G., Gewirtz A.T. (2016). Sex steroid deficiency-associated bone loss is microbiota dependent and prevented by probiotics. J. Clin. Investig..

[32] Nima M.-N., Younes G., Mohammad Hossein D., Pedram T., Farhad K., Ahmad G. (2019). Supportive role of probiotic strains in protecting rats from ovariectomy-induced cortical bone loss. Probiotics Antimicro..

[33] Wang J.H., Wang Y.Y., Gao W.J., Wang B., Zhao H.P., Zeng Y.H., Ji Y.H., Hao D.J. (2017). Diversity analysis of gut microbiota in osteoporosis and osteopenia patients. Peer J..

[34] Wang F., Yu P., Gui X., Wang Y., Xue C., Wang J. (2016). Siaglycoprotein isolated from the eggs of Carassius Auratus prvents bone loss: An efffect associated with the regulation of gut microbiota in ovariectomized rats. Food Funct..

[35] Jin G., Asou Y., Ishiyama K., Okawa A., Kanno T., Niwano Y. (2018). Proanthocyanidin-rich grape seed extract modulates intestinal microbiota in ovariectomized mice. J. Food Sci..

[36] Li C., Huang Q., Yang R., Dai Y., Zeng Y., Tao L., Li X., Zeng J., Wang Q. (2019). Gut microbiota composition and bone mineral loss—epidemiologic evidence from individuals in Wuhan, China. Osteoporos. Int..

[37] Romero Barco C.M., Manrique Arija S., Rodríguez Pérez M. (2012). Biochemical markers in osteoporosis: Usefulness in clinical practice. Reumatol. Clín. Engl. Ed..

[38] Feng W., Ao H., Peng C. (2018). Gut microbiota, short-chain fatty acids, and herbal medicines. Front. Pharmacol..

[39] Corrêa R.O., Fachi J.L., Vieira A., Sato F.T., Vinolo M.A. (2016). Regulation of immune cell function by short-chain fatty acids. Clin. Transl. Immunol..

[40] Li L.S., Rao S.T., Cheng Y.Z., Zhuo X.Y., Deng C.H., Xu N.N., Zhang H., Yang L. (2018). Microbial osteoporosis: The interplay between the gut microbiota and bone via host metabolism and immunity. Microbiology.

[41] Hiroshi T. (2007). Osteoimmunology: Shared mechanisms and crosstalk between the immune and bone systems. Nat. Rev. Immunol..

[42] Lucas S., Omata Y., Hofmann J., Böttcher M., Iljazovic A., Sarter K., Albrecht O., Schulz O., Krishnacoumar B., Krönke G. (2018). Short-chain fatty acids regulate systemic bone mass and protect from pathological bone loss. Nat. Commun..

[43] Jia X., Jia L., Mo L., Yuan S., Zheng X., He J., Chen V., Guo Q., Zheng L., Yuan Q. (2019). Berberine ameliorates periodontal bone loss by regulating gut microbiota. J. Dent. Res..

[44] Kleesen B., Stoof G., Proll J., Schmiedl D., Noack J., Blaut M. (1997). Feeding resistant starch affects fecal and cecal microflora and short-chain fatty acids in rats. J. Anim. Sci..

[45] Beers-Schreurs H.M.G.v., Nabuurs M.J.A., Vellenga L., Kalsbeek-van-der Valk H.J., Wensing T., Breukink H.J. (1998). Weaning and the weanling diet influence the villous height and crypt depth in the small intestine of pigs and alter the concentrations of short-chain fatty acids in the large intestine and blood. J. Nutr..

[46] Oliphant K., Emma A.-V. (2019). Macronutrient metabolism by the human gut microbiome: Major fermentation by-products and their impact on host health. Microbiome.

[47] Li S., You J., Wang Z., Liu Y., Wang B., Du M., Zou T. (2021). Curcumin alleviates high-fat diet-induced hepatic steatosis and obesity in association with modulation of gut microbiota in mice. Food Res. Int..

