# Supplementary material for: Prenylated Isoflavonoids-Rich Extract of Erythrinae Cortex Exerted Bone Protective Effects by Modulating Gut Microbial Compositions and Metabolites in Ovariectomized Rats
Source: Nutrients. 2021 Aug 25;13(9):2943. doi: 10.3390/nu13092943 (PMC8471919; doi:10.3390/nu13092943)
Supplement: Supplementary file 1 [file nutrients-13-02943-s001.zip › nutrients-1326974-supplementary.pdf]

Table S1. The summary of the isolated flavonoids from *Erythrina variegate* L. obtained by literature research

No	Name	Structure	Reference
1	Orientanol B	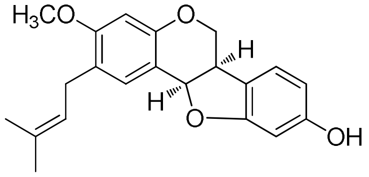	[1]
2	Euchrenone b_{10}	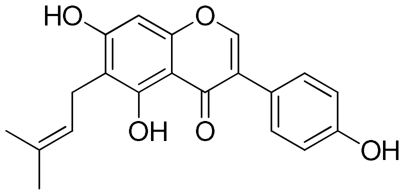	[1]
3	Erythrinin B	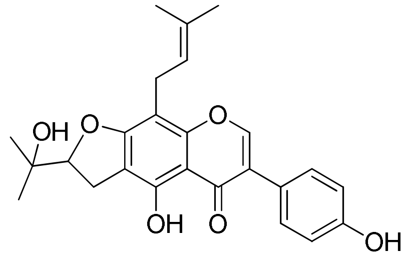	[1]
4	Eryvarins A	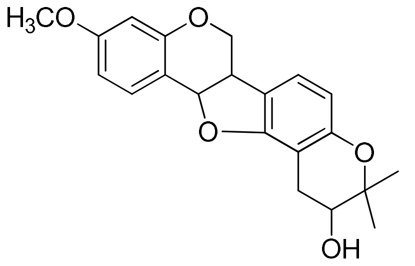	[1]
5	Eryvarins B	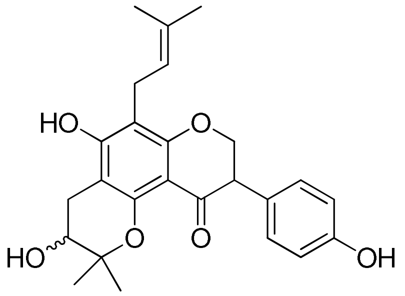	[1]
6	Eryvarins C	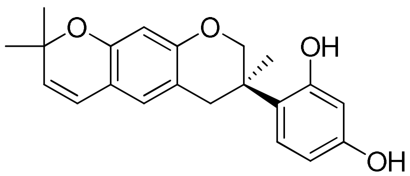	[1]

7	Eryvarins D	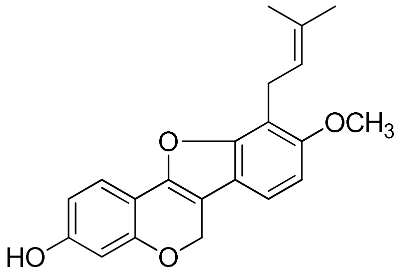	[1]
8	Eryvarins E	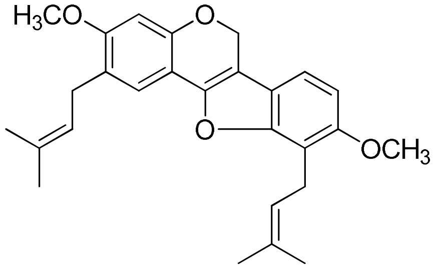	[1]
9	Scandenolone	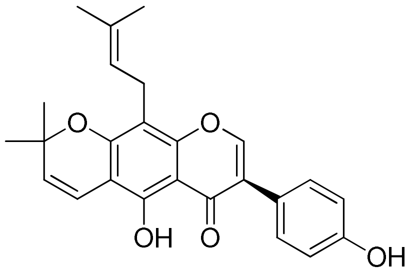	[1]
10	Phaseollin	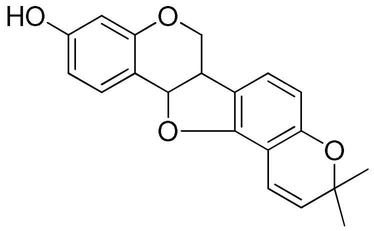	[1]
11	Glabranine	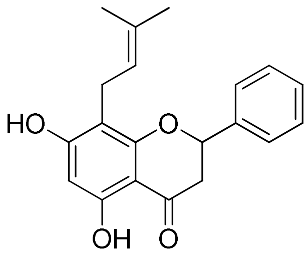	[1]
12	Alpinum isflavone	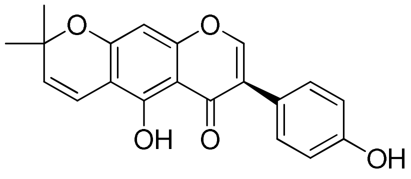	[1]

13	6,8-diprenylgenistein	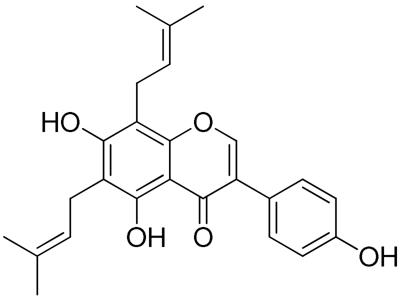	[1]
14	5,4′-dihydroxy-2′-methoxy-8-(3,3-dimethylallyl)-2,2–dimethylpyrano[5,6:6,7]isoflavanone	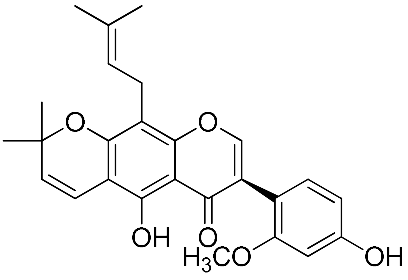	[1]
15	Autriculatin	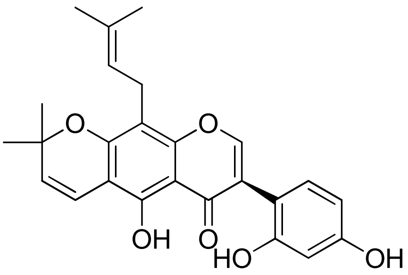	[1]
16	Osajin	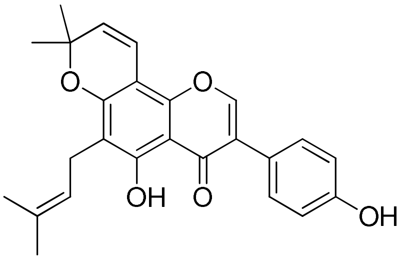	[1]
17	Isoerysenegalensein E	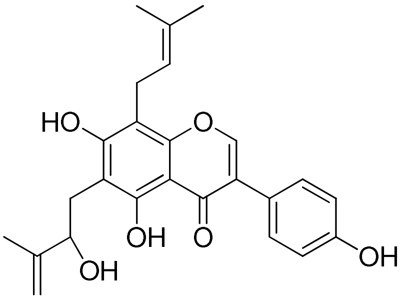	[1]
18	Wighteone	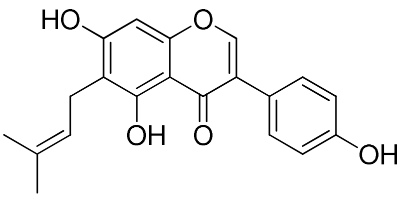	[1]

19	Labumetin	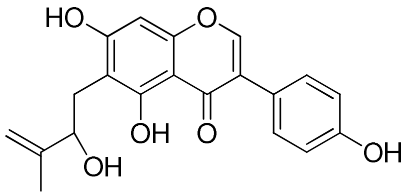	[1]
20	Lupiwighteone	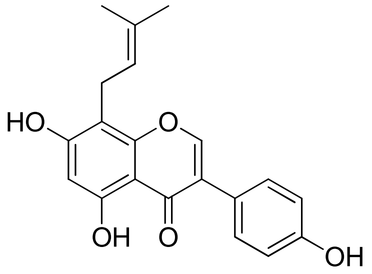	[1]
21	5,4'-dihydroxy-8-(3,3-dimethylally)-2″-methoxyisoporpylufrnao[4,5:6,7]isoflavone	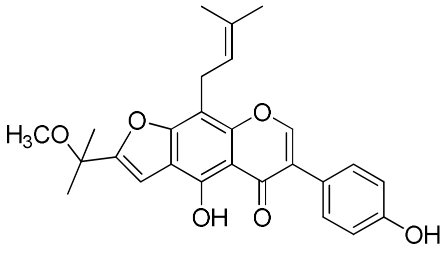	[1]
22	Senegalensin	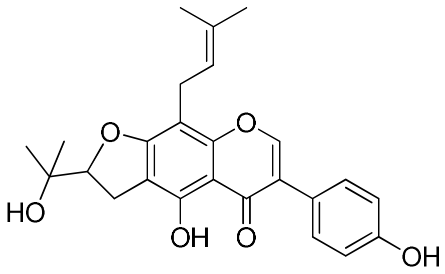	[1]
23	Abyssinone V	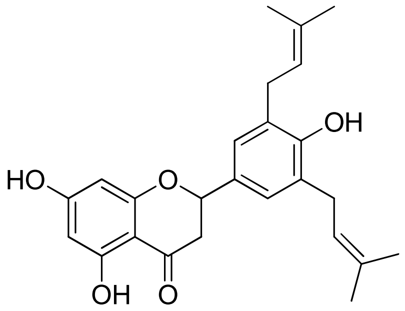	[2]
24	2,3dihydro-5,7-dihydroxy-2-[4-hydroxy-3.5-bis(3-methyl-2-2buten-1-yl)-*4H*-benzopyran-4-one	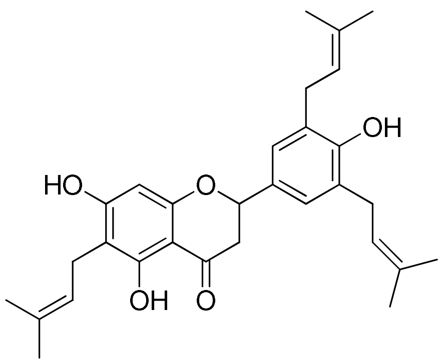	[2]

25	4'-hydroxy-6,3',5'-triperny–l flanonone	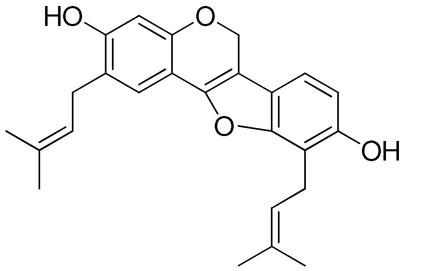	[2]
26	Orientanol B	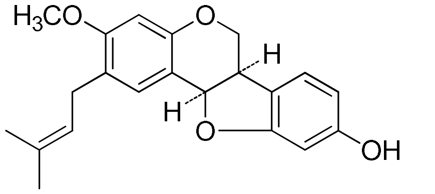	[3]
27	Erystagallin A	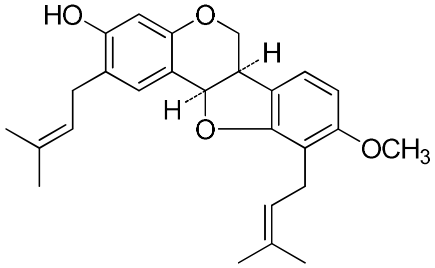	[3]
28	Cristacarpin	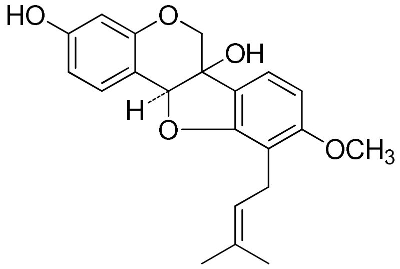	[3]
29	Sigmoidin K	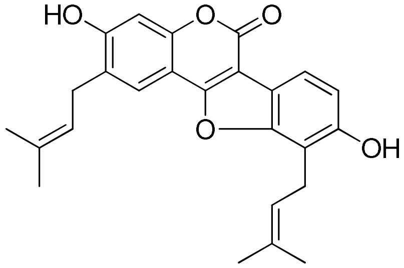	[3]
30	Erycristagallin	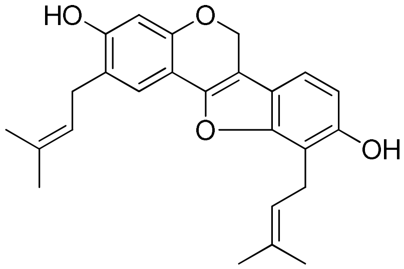	[3]

31	2-(γ,γ-dimethylallyl)-6a-hydroxyphaseollidin: 3,6a,9-trihydroxy2,10-di(γ,γ-dimethylallyl)pterocarpa	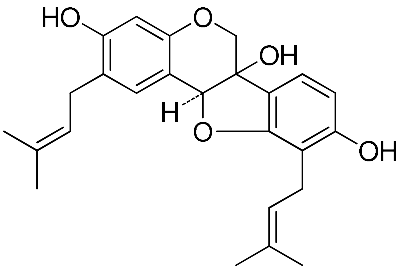	[3]
32	Eryvarin A	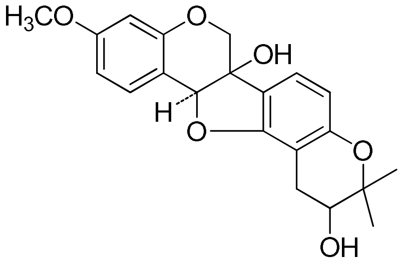	[3]
33	Eryvarins M	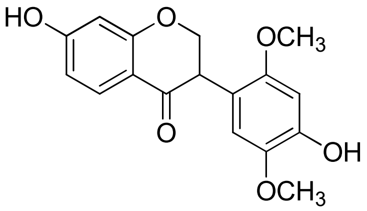	[4]
34	Eryvarins N	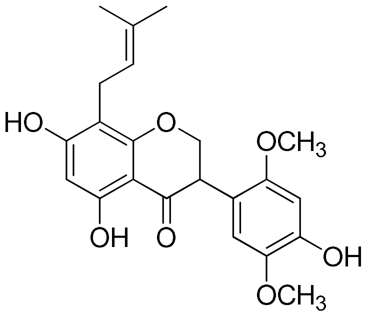	[4]
35	Eryvarins O	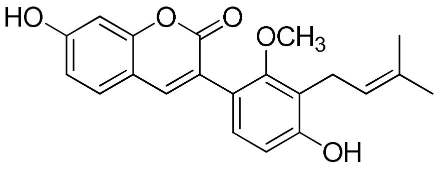	[4]
36	Warangalone	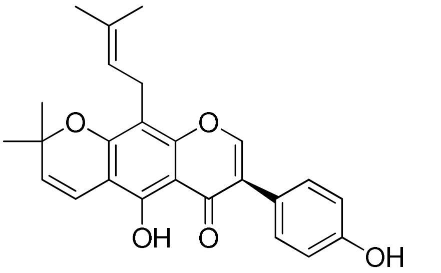	[5]
37	Daidzein7-O-β-D-glucopyranoside	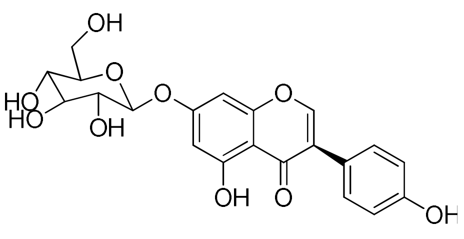	[5]

38	5,4-dihydroxy-8-(3,3-dimethylallyl)-2-methoxyisopropylfurano[4,5:6,7]isoflavone	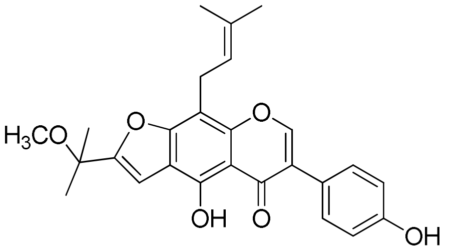	[6]
39	5,7,4′-trihydroxy-6-(3,3-dimethylallyloxiranylmethyl) isoflavone	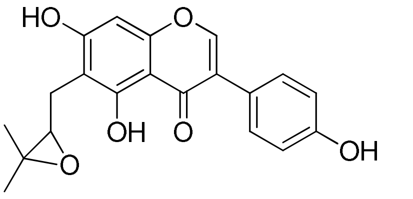	[6]
40	5,4′-dihydroxy-8-(3,3-dimethylallyl)-2-hydroxymethyl-2-methylpyrano[5,6:6,7] isoflavone	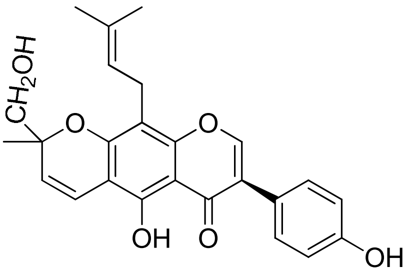	[6]
41	5,4-dihydroxy-2-methoxy-8-(3,3-dimethylallyl)-2,2-dimethylpyrano [5,6:6,7]isoflavanone	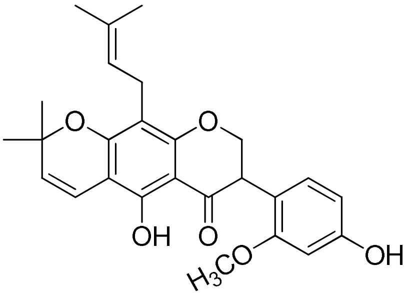	[6]
42	Erycristgallin	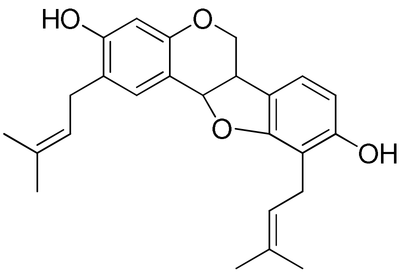	[7]
43	6-Hydroxygenistein	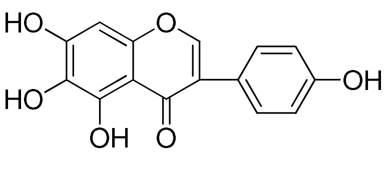	[7]
44	Erythrinins A	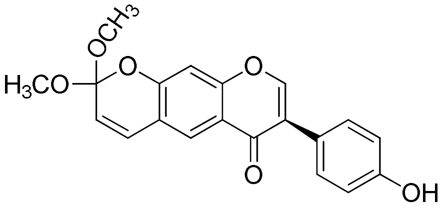	[7]

45	Erythrinins B	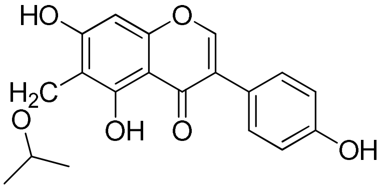	[7]
46	Orientanol B	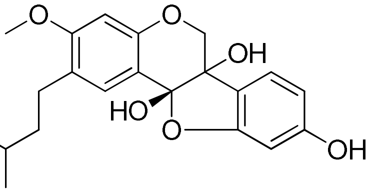	[7]
47	Erystagallin A	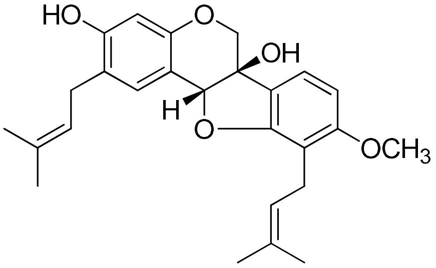	[7]
48	Erythrinins C	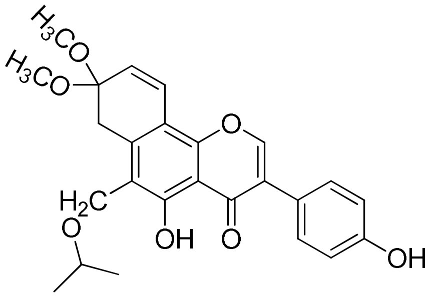	[7]
49	Dihydrofolinin	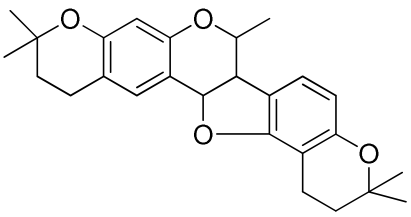	[7]
50	Bidwillon B	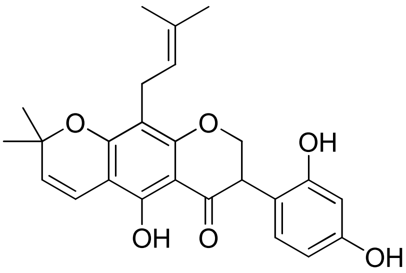	[8]

51	3-(2,4-dihydroxy phenoxy)-7-hydroxy-6,8-di(3,3-dimethylallyl) chromen-4-one	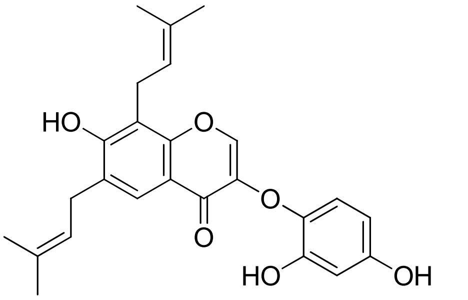	[9]
52	3-(2,4-dihydroxyphenoxy)-7-hydroxy-6,8-di(3,3-dimethylallyl)chromen-4-one	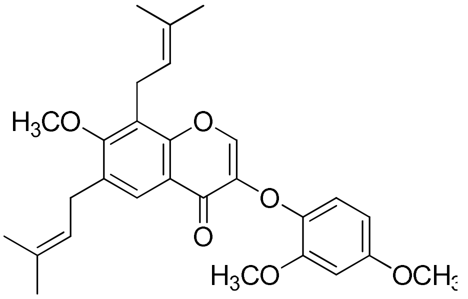	[9]
53	3-(2,4-dihydroxyphenoxy)-8-(3,3-dimethylallyl)-2,2-dimethylpyrano [5,6:6,7]chromen-4-one	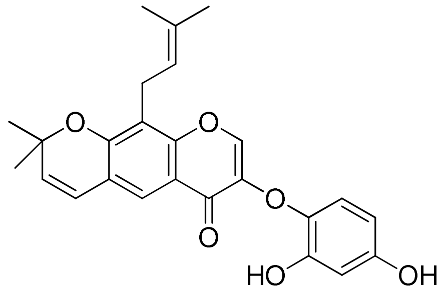	[9]
54	7-(2,4-dihydroxyphenyl)-7,8-dihydro-7-hydroxy-2,2-dimethyl-10-(3-methylbut-2-en-1-yl)-2H,6H-benzo[1,2-b:5,4-b′]dipyran-6-one	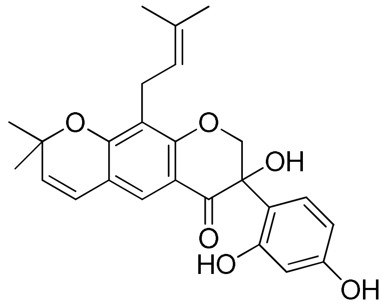	[10]
55	4,10-bis(3-methylbut-2-en-1-yl)-6H-benzofuro[3,2-c][1]benzopyran-3,9-diol	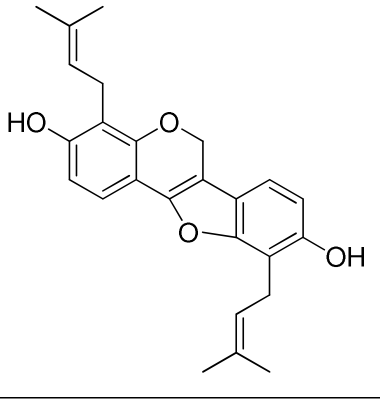	[10]

56	3,7-dihydroxy-6,8-bis(3-methylbut-2-en-1-yl)-4H-1-benzopyran-4-one	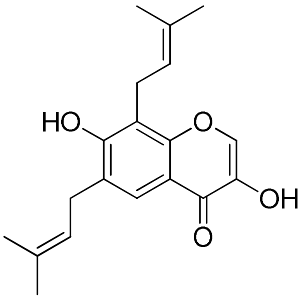	[10]
57	Eryvarins S	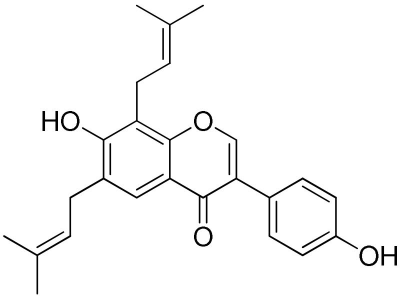	[11]
58	Eryvarins T	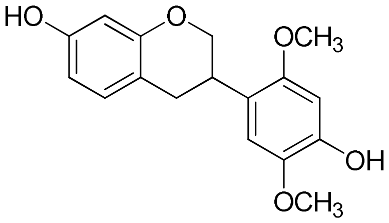	[11]
59	Eryvarin U	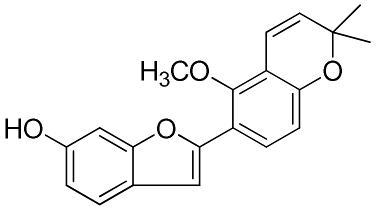	[11]
60	Eryvarins H	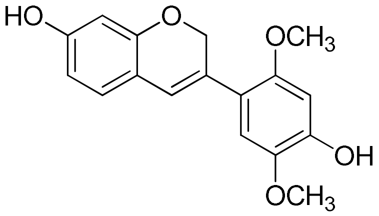	[12]
61	Eryvarins C	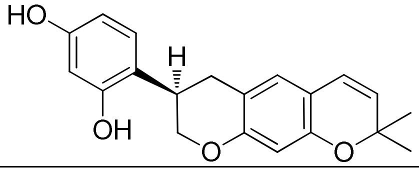	[13]

62	Eryvarins E	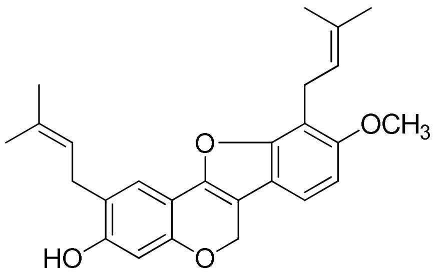	[13]
63	Eryvarin B	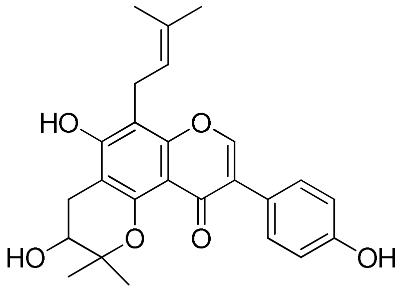	[14]

Table S2. Identified prenylated isoflavonoids from EC extract by ESI-Orbitrap-MS analysis

Retention Time	Neutral mass	Theoretical [M+H]+	Measured mass	Mass error	Fragments	Possible Metabolite
10.06	338.1154	339.1227	339.1223	1.18	>321.1118, 267.0649	Euchrenone b10
11.65	352.1311	353.1384	353.1383	0.28	>163.0391	Eryvarins O
11.69	370.1416	371.1489	371.1488	0.27	>353.1380, 315.0862	Eryvarin A
12.00	354.1103	355.1176	355.1171	1.41	>283.0596, 337.1063	5,4'-dihydroxy-8-(3,3-dimethylally)-2″-methoxyisoporpylufrnao[4,5:6,7]isoflavone
12.01	422.1729	423.1802	423.1801	0.24	>405.3517, 367.1173, 355.1171	3-(2,4-dihydroxy phenoxy)-7-hydroxy-6,8-di(3,3-dimethylallyl) chromen-4-one
12.48	338.1154	339.1227	339.1223	1.18	>283.0596	5,4'-dihydroxy-2'-methoxy-8-(3,3-dimehtylally-2″,2″-dimethylpyrano[5,6:6,7] isoflavanone
13.08	322.1205	323.1278	323.1275	0.93	>267.0649	Phaseollin
13.54	354.1467	355.154	355.154	0.00	>327.1588, 299.0910, 231.1014, 271.0963	Cristacarpin
13.59	336.1362	337.1435	337.143	1.48	>281.0805	Eryvarins D
13.75	438.1679	439.1752	439.1746	1.37	>421.1640	Erythrinins C
13.85	390.1823	391.1896	391.19	1.02	>335.1653	4,10-bis(3-methylbut-2-en-1-yl)-6H-benzofuro[3,2-c][1]benzopyran-3,9-diol
13.85	390.1831	391.1904	391.1909	1.28	>335.1276, 189.0910	Erycristagallin
13.90	338.1154	339.1227	339.1224	0.88	>283.0598	Wighteone
15.23	422.1729	423.1802	423.1801	0.24	>405.1694, 367.1171, 355.1175	Senegalensin
15.23	422.1729	423.1802	423.1798	0.95	>405.1695, 367.1172, 355.1173	7-(2,4-dihydroxyphenyl)-7,8-dihydro-7-hydroxy-2,2-dimethyl-10-(3-methylbut-2-en-1-yl)-2H,6H-benzo[1,2-b:5,4-b′] dipyran-6-one

15.67	408.1937	409.201	409.1996	3.42	>353.1379	Abyssinone V
15.71	336.0998	337.1071	337.1066	1.48	> 319.0963, 283.0597	Alpinum isflavone
15.87	422.1729	423.1802	423.18	0.47	>367.1171, 355.1172	Isoerysenegalensein E
15.87	354.1103	355.1176	355.1171	1.41	>299.0547	5,7,4-trihydroxy-6-(3,3-dimethylallyloxiranylmethyl) isoflavone
15.87	422.1729	423.1802	423.18	0.47	>367.1171, 355.1172	Erythrinin B
16.28	404.1624	405.1697	405.1692	1.23	>349.1064	Warangalone
16.42	404.1988	405.2061	405.2056	1.23	>349.1430	Eryvarins E
16.49	420.1573	421.1646	421.1645	0.24	>365.1010	5,4-dihydroxy-8-(3,3-dimethylallyl)-2-hydroxymethyl-2-methylpyrano[5,6:6,7]isoflavone
16.95	408.1937	409.201	409.2009	0.24	>353.1979	2-(γ,γ-dimethylallyl)-6a-hydroxyphaseollidin: 3,6a,9-trihydroxy2,10-di(γ,γ-dimethylallyl) pterocarpa
17.13	420.1573	421.1646	421.1636	2.37	>403.1534, 365.1013, 353.1016	3-(2,4-dihydroxyphenoxy)-8-(3,3-dimethylallyl)-2,2-dimethylpyrano [5,6:6,7] chromen-4-one
17.62	390.1831	391.1904	391.19	1.02	>335.1275, 323.1273	Eryvarins S
17.78	434.1729	435.1802	435.1795	1.61	>379.1169	5,4-dihydroxy-2-methoxy-8-(3,3-dimethylallyl)-2,2–dimethylpyrano [5,6:6,7] isoflavanone
17.98	476.2563	477.2636	477.2635	0.21	>421.2006, 365.1382, 219.0651	2,3dihydro-5,7-dihydroxy-2-[4-hydroxy-3.5-bis(3-methyl-2-2buten-1-yl)-*4H*-benzopyran-4-one
18.43	404.1624	405.1697	405.1689	1.97	>349.1064, 337.1067	Sigmoidin K

Supplementary reference

1. Li, X.L. Study of antiosteoporotic constituents of Erytherina variegate L. . Shenyang Pharmaceutical University, 2004.
2. Hegde, V.R.; Dai, P.; Patel, M.G.; Puar, M.S.; Das, P.; Pai, J.; Bryant, R.; Cox, P.A. Phospholipase A2 Inhibitors from an Erythrina Species from Samoa. *J. Nat. Prod* **1997**, *60*, 537-539.
3. Sato, M.; Tanaka, H.; Fujiwara, S.; Hirata, M.; Yamaguchi, R.; Etoh, H.; Tokuda, C. Antibacterial property of isoflavonoids isolated from Erythrina variegata against cariogenic oral bacteria. *Phytomedicine* **2003**, *10*, 427-433.
4. Tanaka, H.; Hirata, M.; Etoh, H.; Sako, M.; Sato, M.; Murata, J.; Murata, H.; Darnaedi, D.; Fukai, T. Six new constituents from the roots of Erythrina variegata. *Chem. Biodivers.* **2004**, *1*, 1101-1108.
5. Talla, E.; Yankep, E.; Tanyi Mbafor, J. Chemical constituents from root barks of Erythrina mildbraedii and stem barks of Erythrina addisoniae. *Bull. Chem. Soc. Ethiop.* **2014**, *28*, 155-159.
6. Li, X.; Wang, N.; Wong Man, S.; Albert, S.C.C.; Yao, X. Four new isoflavonoids from the stem bark of Erythrina variegata. *Chem. Pharm. Bull.* **2006**, *54*, 570-573.
7. Kumar, A.; Lingadurai, S.; Jain, A.; Barman, N.R. Erythrina variegata Linn: A review on morphology, phytochemistry, and pharmacological aspects. *Pharmacogn. Rev.* **2010**, *4*, 147-152.
8. Sato, M.; Tanaka, H.; Yamaguchi, R.; Kato, K.; Etoh, H. Synergistic effects of mupirocin and an isoflavanone isolated from Erythrina variegata on growth and recovery of methicillin-resistant Staphylococcus aureus. *Int. J. Antimicrob. Agents* **2004**, *24*, 241-246.
9. Tanaka, H.; Hirata, M.; Etoh, H.; Shimizu, H.; Sako, M.; Murata, J.; Murata, H.; Darnaedi, D.; Fukai, T. Eryvarins F and G, two 3-phenoxychromones from the roots of Erythrina variegata. *Phytochemistry* **2003**, *62*, 1243-1246.
10. Tanaka, H.; Atsumi, I.; Shirota, O.; Sekita, S.; Sakai, E.; Sato, M.; Murata, J.; Murata, H.; Darnaedi, D.; Chen, I.S. Three new constituents from the roots of Erythrina variegata and their antibacterial activity against methicillin - resistant staphylococcus aureus. *Chem. Biodivers.* **2011**, *8*, 476-482.
11. Tanaka, H.; Sudo, M.; Hirata, M.; Sako, M.; Sato, M.; Chen, I.S.; Fukai, T. Two new isoflavonoids and a new 2 - arylbenzofuran from the roots of Erythrina variegata. *ChemInform* **2005**, *36*, No.
12. Tanaka, H.; Hirata, M.; Etoh, H.; Sako, M.; Sato, M.; Murata, J.; Murata, H.; Darnaedi, D.; Fukai, T. Four new isoflavonoids and a new 2 - arylbenzofuran from the roots of Erythrina variegata. *ChemInform* **2004**, *35*, No.
13. Tanaka, H.; Hirata, M.; Etoh, H.; Watanabe, N.; Shimizu, H.; Ahmad, M.; Anwar, M. Three new isoflavonoids from Erythrina variegata. *Heterocycles* **2001**, *55*, 2341-2347.
14. Tanaka, H.; Etoh, H.; Shimizu, H.; Makita, T.; Tateishi, Y. Two new isoflavonoids from Erythrina variegata. *Planta Med.* **2000**, *66*, 578-579.
